# Urachal inflammatory myofibroblastic tumor: a case report

**DOI:** 10.3389/fonc.2026.1895685

**Published:** 2026-08-11

**Authors:** Jiafu Liu, Ran Wei, Xiaoxing Miao, Jinbo Han, Jie Wang

**Affiliations:** 1 Hebei North University, Zhangjiakou, Hebei, China; 2 Department of Urology, Handan Central Hospital, Handan, Hebei, China

**Keywords:** anaplastic lymphoma kinase, bladder mass, case report, inflammatory myofibroblastic tumor, urachal tumor, urachus

## Abstract

**Background:**

Inflammatory myofibroblastic tumor (IMT) is an uncommon mesenchymal neoplasm. Urachal involvement is exceedingly rare, and evidence regarding its clinical presentation, diagnosis, and management remains limited.

**Case presentation:**

We report the case of a 62-year-old female patient who presented with urinary frequency and dysuria for 8 days. Computed tomography (CT) revealed a mass at the junction of the urachus and the anterosuperior wall of the bladder, which was further confirmed by cystoscopy. The patient underwent laparoscopic resection of the urachal mass combined with partial cystectomy. Histopathological examination of the surgical specimen confirmed IMT, and immunohistochemical staining was positive for anaplastic lymphoma kinase (ALK) and vimentin. At the 3-month postoperative follow-up, CT and cystoscopy showed no evidence of local recurrence.

**Conclusion:**

UIMT should be considered in the differential diagnosis of urachal or anterosuperior bladder-wall masses. Definitive diagnosis requires integrated histopathological and immunohistochemical assessment, and complete resection with negative margins is the preferred treatment for localized, resectable disease.

## 1 Introduction

Inflammatory myofibroblastic tumor (IMT) is a rare mesenchymal neoplasm with intermediate biological behavior between benign and malignant tumors (1). Most IMTs arise in the lung, abdomen, or pelvis and usually remain localized, with distant metastasis reported in fewer than 5% of cases (2). Urachal IMT is extremely rare, and reliable incidence estimates are unavailable because the published evidence is largely limited to individual case reports and small series (3–6). Its etiology remains unclear. The clinical presentation of UIMT depends on its location, size, depth of bladder-wall invasion, and involvement of adjacent perivesical structures (7). When UIMT invades or compresses the bladder, patients may present with hematuria, dysuria, urinary frequency, or other bladder-irritation symptoms (7). In this report, we describe the clinical course of a 62-year-old female patient diagnosed with this extremely rare form of UIMT.

## 2 Case report

**Figure 1:**
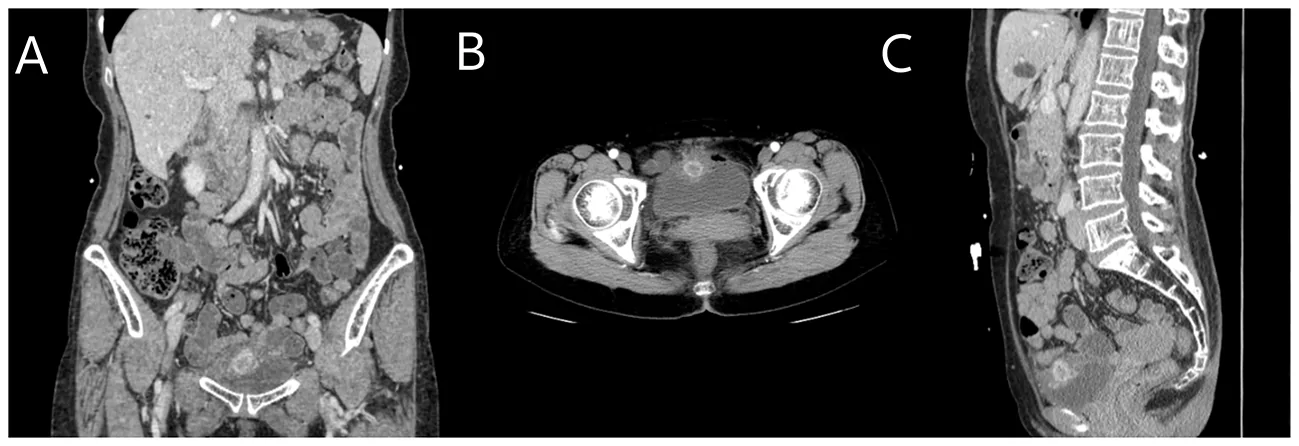
Preoperative contrast-enhanced CT of urachal inflammatory myofibroblastic tumor. **(A)** Coronal view; **(B)** Axial view; **(C)** Sagittal view. A well-defined soft-tissue mass was located in the midline of the urachal region.

A 62-year-old woman presented with dysuria and urinary frequency for 8 days and was evaluated for a suspected bladder neoplasm. Ultrasonography and computed tomography (CT) revealed a broad-based mass measuring 3.0 × 1.5 × 2.0 cm at the junction of the urachus and the anterosuperior bladder wall, protruding into the bladder lumen (**Figure 1**). She had no history of smoking or alcohol use and no known infectious or hereditary disorders.

The patient underwent laparoscopic excision of the urachal mass combined with partial cystectomy. A 3-cm supraumbilical midline incision and two additional lateral ports were used to establish laparoscopic access. Intraoperatively, a papillary mass approximately 3 cm in diameter was identified at the junction of the urachus and the anterior bladder wall. The urachal mass, adjacent soft tissue, and a cuff of the involved anterior bladder wall were removed en bloc with an approximately 1-cm gross circumferential margin. The umbilicus was preserved and was not included in the resection. The bladder defect was closed with 2–0 absorbable barbed sutures, and a pelvic drain and urinary catheter were left in place. Gross examination showed a surgical specimen measuring 5.5 × 5.0 × 2.0 cm, consisting of the urachal mass with attached bladder wall and adjacent soft tissue. The tumor measured approximately 2.0 × 1.5 × 1.5 cm.

**Figure 2:**
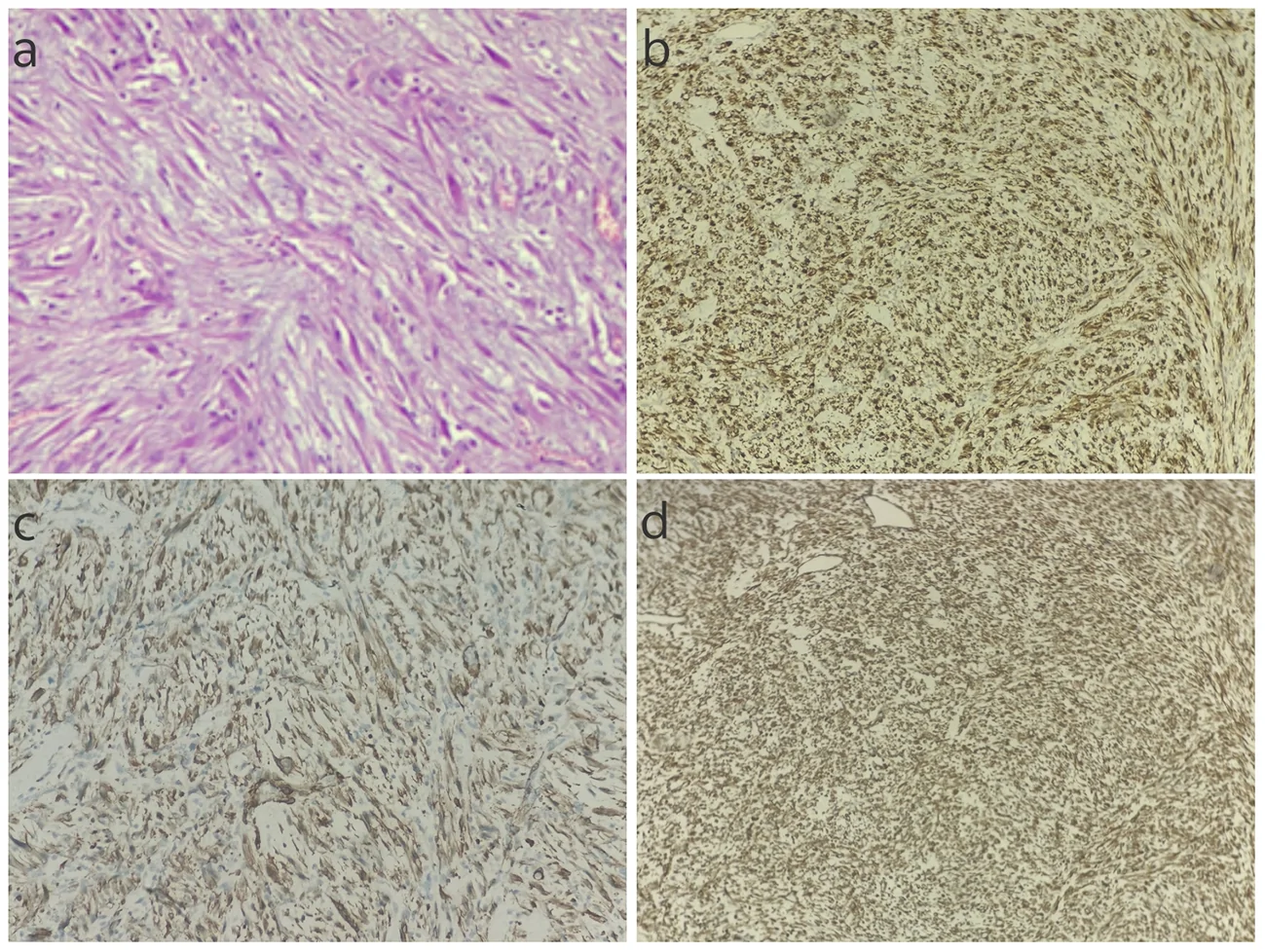
Histopathological and immunohistochemical findings of the urachal tumor. **(A)** H&E staining shows spindle-cell proliferation arranged in intersecting fascicles within a collagenized stroma, accompanied by scattered inflammatory cells. **(B–D)** Tumor cells show positive immunostaining for ALK, SMA, and vimentin, respectively.

Histopathological examination showed a spindle-cell proliferation arranged in intersecting fascicles within a variably collagenized stroma, accompanied by scattered inflammatory cells and focal necrosis (**Figure 2A**). Marked edema of the bladder mucosa and urothelial hyperplasia were also present. The circumferential and basal resection margins were histologically free of tumor, and the tumor-to-lateral surgical margin distance was 1.8 cm. Immunohistochemical staining showed that the tumor cells were positive for vimentin, SMA, and ALK (**Figures 2B–D**), while desmin and S-100 were negative. DOG1 and Bcl-2 were also negative. STAT6 was reported as positive, although its subcellular localization was not documented. CD117 showed focal reactivity of uncertain cellular origin, CD34 highlighted vascular structures rather than tumor cells, and beta-catenin showed membranous staining without documented nuclear expression. The Ki-67 labeling index was approximately 5%.

The patient’s urinary frequency and dysuria resolved by postoperative day 7, and she was subsequently discharged. Follow-up computed tomography performed 3 months after surgery showed no evidence of recurrence, and the patient reported no significant pain. Regular follow-up was recommended, and the patient was advised to seek further evaluation if urinary symptoms recurred.

## 3 Discussion

Published evidence on UIMT remains limited. Selected published UIMT reports with case-level clinical data are summarized individually in **Table 1** (3–6, 8–13). The molecular basis of UIMT remains incompletely characterized. ALK rearrangement has been confirmed by fluorescence *in situ* hybridization in a reported UIMT, and an FN1::ALK fusion has been identified by next-generation sequencing in another case. Broader molecular studies of IMTs have identified recurrent kinase fusions involving ALK, ROS1, and RET, supporting a neoplastic basis in molecularly defined subsets of these tumors (8, 9, 14, 15).

**Table 1:** Selected case-by-case summary of published urachal inflammatory myofibroblastic tumor reports.

Source/year	Age/sex	Presentation and tumor features	Treatment	ALK/molecular findings	Follow-up and outcome
George et al., 2021 (3)	27-year-old female	Lower abdominal/pelvic pain and dysuria; 3.2-cm solid-cystic mass superior and anterior to the bladder	Robot-assisted wide excision of the urachus and involved adjacent tissues with partial cystectomy	ALK positive; ALK rearrangement confirmed	Asymptomatic with no recurrence at 5 months
Liu et al., 2025 (4)	38-year-old female	Lower abdominal mass and discomfort during the second trimester of pregnancy; hypervascular solid mass at the urachal-anterior bladder-wall junction; 2.0-cm intravesical component after delivery	Observation during pregnancy, followed by laparoscopic extended partial cystectomy 50 days after vaginal delivery	ALK status and molecular testing NR	No recurrence or metastasis; follow-up duration NR
Liu and Bai, 2026 (5)	12-year-old male	Dysuria and gross hematuria; urachal mass with bladder invasion	Laparoscopic urachal mass resection and partial cystectomy	ALK positive	No recurrence or metastasis at 6 months
Venkatesh and Madhusudhan, 2016 (6)	50-year-old male	Dysuria and hematuria; 3.8-cm anterosuperior bladder mass extending toward the umbilicus	Complete excision with partial cystectomy	ALK positive	No recurrence at 3 years
Nascimento et al., 2004 (8)	10-year-old male	Lower abdominal pain; 3.5-cm cystic mass superior and anterior to the bladder	Excision with resection of the bladder dome	ALK positive; ALK rearrangement confirmed by FISH	NR
Tara et al., 2024 (9)	12-year-old male	Lower abdominal pain and burning micturition; 2.4-cm mass anterior to the bladder wall	Laparoscopic tumor excision	FN1::ALK fusion identified by NGS	NR
Meyer et al., 2000 (10)	3-year-old female	Bladder mass associated with a residual urachus; presenting symptom NR	Complete excision with partial cystectomy	NR	NR
Tunca et al., 2006 (11)	16-year-old male	Lower abdominal pain, hematuria, and dysuria; 10-cm urachal mass mimicking invasive bladder carcinoma	Complete excision with partial cystectomy	SMA negative; ALK or molecular testing NR	NR
Wang et al., 2018 (12)	77-year-old female	Lower abdominal mass; 10-cm suprapubic mass with central necrosis and hemorrhage	Laparoscopic radical urachal resection and partial bladder resection	ALK negative; SMA and vimentin positive	No recurrence or metastasis at 22 months
Cui et al., 2024 online/2025 print (13)	65-year-old male	Presenting symptoms NR; diffuse-sheet lesion in the muscularis propria of the urachal submucosa with infiltration into surrounding smooth muscle	Surgical resection; operative details NR	ALK status and molecular testing NR	NR
Present case	62-year-old female	Urinary frequency and dysuria; mass at the urachal-anterosuperior bladder-wall junction	Laparoscopic en bloc excision of the urachal mass, adjacent soft tissue, and involved bladder-wall cuff; umbilicus preserved	ALK immunoreactive; FISH/NGS not performed	No local recurrence at 3 months

This table presents selected reports with case-level clinical information. NR indicates data unavailable from the published record consulted. ALK, anaplastic lymphoma kinase; FISH, fluorescence *in situ* hybridization; NGS, next-generation sequencing; NR, not reported; SMA, smooth muscle actin; UIMT, urachal inflammatory myofibroblastic tumor.

The clinical and imaging manifestations of UIMT are nonspecific and depend on lesion location and bladder involvement. Patients may present with hematuria, dysuria, urinary frequency, or pelvic pain. CT and cystoscopy are useful for defining the relationship of the lesion to the urachus and bladder wall, but definitive diagnosis requires pathological evaluation (7, 16–20).

The diagnosis of UIMT should be based on integrated morphology and immunophenotype. Typical findings include spindle myofibroblastic cells in myxoid or collagenous stroma with a mixed inflammatory infiltrate; SMA and vimentin are commonly expressed, and ALK is positive in a subset of tumors (6, 21, 22). In the present case, the spindle-cell morphology, inflammatory background, SMA and vimentin expression, and ALK immunoreactivity supported IMT. Solitary fibrous tumor was considered because STAT6 was reported as positive, although the subcellular staining pattern was not documented and CD34 highlighted vascular structures rather than tumor cells. Gastrointestinal stromal tumor was not favored because DOG1 was negative and focal CD117 reactivity was nonspecific. Desmoid-type fibromatosis was also considered, but beta-catenin showed membranous rather than documented nuclear expression. The original pathology report did not include a formal mitotic count, assessment of atypical mitoses, or characterization of the type and extent of necrosis. It also did not document the subcellular localization of ALK or STAT6 staining or the cellular source of focal CD117 reactivity. ALK rearrangement was not evaluated by fluorescence *in situ* hybridization or next-generation sequencing; thus, ALK immunoreactivity was considered supportive but did not provide molecular confirmation of an ALK-rearranged tumor.

## 4 Conclusion

This case adds to the limited clinicopathological evidence on UIMT and illustrates its potential to mimic a primary bladder neoplasm when arising at the urachal–bladder junction. Correlation of the anatomical origin with spindle-cell morphology and immunophenotypic findings was central to establishing the diagnosis. Negative-margin resection was achieved, although the 3-month follow-up permits only an early assessment of the postoperative course.

## Data Availability

The original contributions presented in the study are included in the article/supplementary material. Further inquiries can be directed to the corresponding author.
